# Improved Aptamers for the Diagnosis and Potential Treatment of HER2-Positive Cancer

**DOI:** 10.3390/ph9020029

**Published:** 2016-05-19

**Authors:** Marlies Gijs, Gregory Penner, Garth B. Blackler, Nathalie R.E.N. Impens, Sarah Baatout, André Luxen, An M. Aerts

**Affiliations:** 1 Radiobiology Unit, Belgian Nuclear Research Centre (SCK•CEN), 2400 Mol, Belgium; nimpens@sckcen.be (N.R.E.N.I.); sbaatout@sckcen.be (S.B.); aaerts@sckcen.be (A.M.A.); 2 Cyclotron Research Centre, University of Liège, 4000 Liège, Belgium; aluxen@ulg.ac.be; 3 NeoVentures Biotechnology Inc., London, N6A 1A1 ON, Canada; gpenner@neoventures.ca (G.P.); gblackler@neoventures.ca (G.B.B.)

**Keywords:** aptamer, DNA, HER2, diagnosis, therapeutics, cancer

## Abstract

Aptamers provide a potential source of alternative targeting molecules for existing antibody diagnostics and therapeutics. In this work, we selected novel DNA aptamers targeting the HER2 receptor by an adherent whole-cell SELEX approach. Individual aptamers were identified by next generation sequencing and bioinformatics analysis. Two aptamers, HeA2_1 and HeA2_3, were shown to bind the HER2 protein with affinities in the nanomolar range. In addition, both aptamers were able to bind with high specificity to HER2-overexpressing cells and HER2-positive tumor tissue samples. Furthermore, we demonstrated that aptamer HeA2_3 is being internalized into cancer cells and has an inhibitory effect on cancer cell growth and viability. In the end, we selected novel DNA aptamers with great potential for the diagnosis and possible treatment of HER2-positive cancer.

## 1. Introduction

One molecular target with high potential for targeted cancer therapy is the human epidermal growth factor receptor 2 (HER2, also known as ErbB2). HER2 gene amplification and protein overexpression is present in 15% to 20% of all breast cancers, and is associated with aggressive disease and poor patient prognosis [1,2]. In addition, HER2 overexpression is associated with resistance to certain chemotherapeutics [3], an increased risk for brain metastases [4] and higher recurrence of the disease [5]. Trastuzumab (Herceptin^®^, Genentech), a humanized monoclonal antibody targeting HER2, has been approved for the treatment of HER2 positive early-stage breast cancer, metastatic breast cancer and metastatic cancer of the stomach or gastroesophageal junction [6]. However, only one third of patients respond to trastuzumab therapy and most of the responders eventually relapse [7]. Moreover, resistance to trastuzumab is of major concern [8] and heart failure occurred in 1% to 4% of patients treated with trastuzumab [9]. Hence, there is a need for novel therapeutic agents.

Aptamers provide a potential source of such alternative targeting molecules for existing antibody therapeutics such as trastuzumab. Aptamers are short, non-coding, single-stranded oligonucleotides (DNA or RNA) that mimic antibodies in their ability to bind to a specific target. Aptamers are promising agents for therapeutic use as they are easily and inexpensively synthesised, poorly immunogenic and non-toxic (as reviewed in [10,11]). Aptamers have numerous valuable applications in cancer research, including biomarker discovery, *in vitro* and *in vivo* diagnostics (reviewed in [12]). In addition, aptamers are being developed as therapeutics agents for the treatment of a wide range of cancers via aptamer-mediated delivery of therapeutic payloads (such as drugs, siRNA and radionuclides (reviewed in [13,14]) or via effects on target function (reviewed in [11,15,16,17,18,19]).

Typically, aptamers are generated by an *in vitro* selection process known as systematic evolution of ligands by exponential enrichment (SELEX) [20,21,22]. This process allows the identification of aptamers from a large pool of random sequences or library. During the process, aptamers are subjected to iterative rounds of selection, separation and amplification. As SELEX is an evolutionary affinity-driven process, specific sequences (which bind in a specific way) will dominate non-specific sequences (which bind at random). The most crucial step of the SELEX process is achieving the appropriate balance of stringency in the separation of the bound and unbound sequences. Too much stringency introduces the risk of losing the best aptamers, while too little stringency may lead to ineffective selection for good aptamers.

Surface proteins, such as the HER2 receptor, are highly accessible drug targets and therefore are central in targeted therapy. The majority of aptamers targeting HER2 reported so far have been selected using the purified, soluble portion of the exo-cellular domain of the HER2 protein. However, these domains alone may exhibit different conformations or lack post-translational modifications thus altering their epitope potential. Hence, SELEX using living cells (called whole-cell SELEX) is preferred. The use of adherent cells (as monolayers in cultured dishes) is favorable because it allows easy separation of bound and unbound sequences during selection and easy removal of dead cells (which generally have high affinity for nucleic acids) [23]. We describe herein a whole-cell SELEX process using adherent SKBR3 breast cancer cells.

In order to avoid the drawbacks seen with long aptamers (higher production cost with lower yields and purity) and RNA aptamers (increased susceptibility to nuclease degradation), we focused on short DNA aptamers. For this, we used the Dubbles concept designed by NeoVentures Biotechnology Inc. [24]. This concept involves the separation of the PCR-amplified double-stranded DNA amplicon into single-strands by heating to 95 °C for 10 min and then “snap-cooling” the resulting single-strands immediately for 15 min. First, this helps to stabilize the secondary and tertiary structures of the single-stranded sequences in favour of double-stranded annealing. Second, re-annealing of the single-stranded sequences is driven by the homology of the primer regions rather than complementary strands. As a result, heteroduplexes (called Dubbles) are formed which allow interactions of the single-stranded random regions with the target. This concept is advantageous because it avoids the hybridization of primer sequences onto complementary parts of the internal random region (which may make this region unavailable for binding to the target) [25], the involvement of primer sequences in binding to the target [26,27], and the need for complex techniques to separate double-stranded to single-stranded DNA after PCR amplification [28]. At the end, the internal random regions of the selected aptamers are chemically synthesized without the primer regions, which results in short(er) aptamers. Several aptamers have been selected using this strategy, for example aptamers targeting aflatoxin [29] and ochratoxin A [30]. We have used a library containing a 40-mer random region and, thanks to the Dubbles technology, generated aptamers of this length (average 12 kDa).

Lately, high throughput next generation sequencing (NGS) and bioinformatics are preferred over traditional cloning and subsequent sequencing as it avoids the need for a high number of iterative selection rounds while reducing time, PCR bias and artefacts [25,31,32,33,34]. We included NGS of every selection round and subsequent identification of the best aptamer sequences from the selected aptamer pools based on frequency of the individual aptamer sequences within the final selected aptamer pool (abundance) and the rate at which these frequencies were increasing over multiple selection rounds (enrichment).

Several aptamers targeting HER2 have been reported. Most of these aptamers are RNA aptamers [35,36,37] and considerably longer (>71-mer) [35,36,37,38,39]. In addition, they were selected using (a part of) the purified HER2 protein [36,38,39,40,41] or using a mouse cell line [37]. Moreover, the majority of these studies did not utilize next generation sequencing to probe the selected libraries deeply [35,36,39,40,41]. Finally, only few aptamers targeting HER2 were tested for their affinity and specificity. 

In this study, we isolated novel short (40-mer) HER2-specific DNA aptamers from whole-cell SELEX using adherent human breast cancer cells (SKBR3). The selected aptamers were further characterized in terms of binding properties and potential inhibitory effect on cell growth.

## 2. Results and Discussion

### 2.1. Whole-Cell SELEX on SKBR3 Breast Cancer Cells

**Figure 1:**
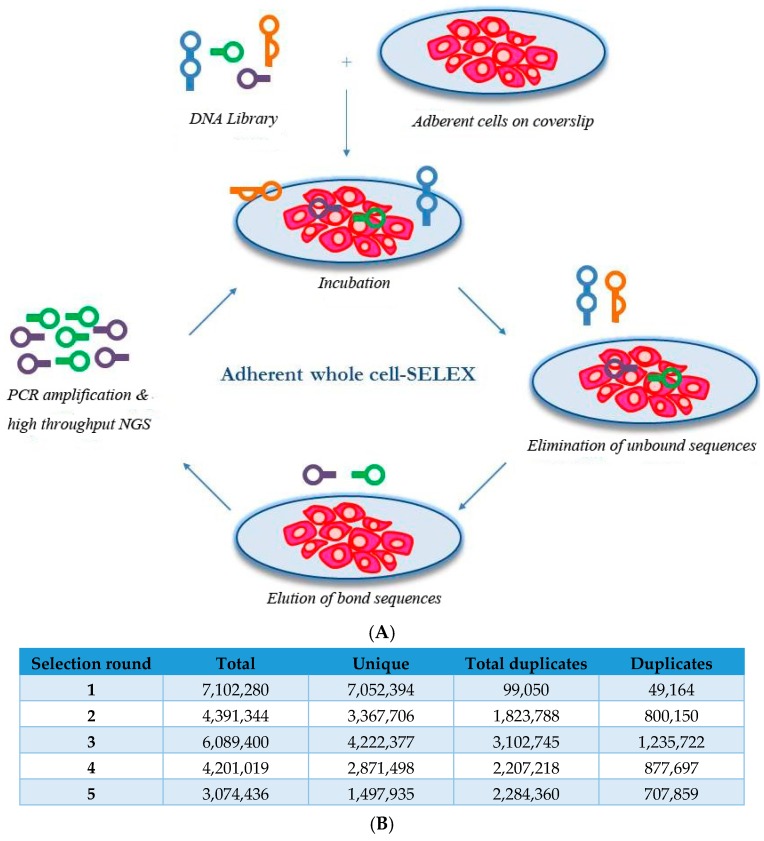

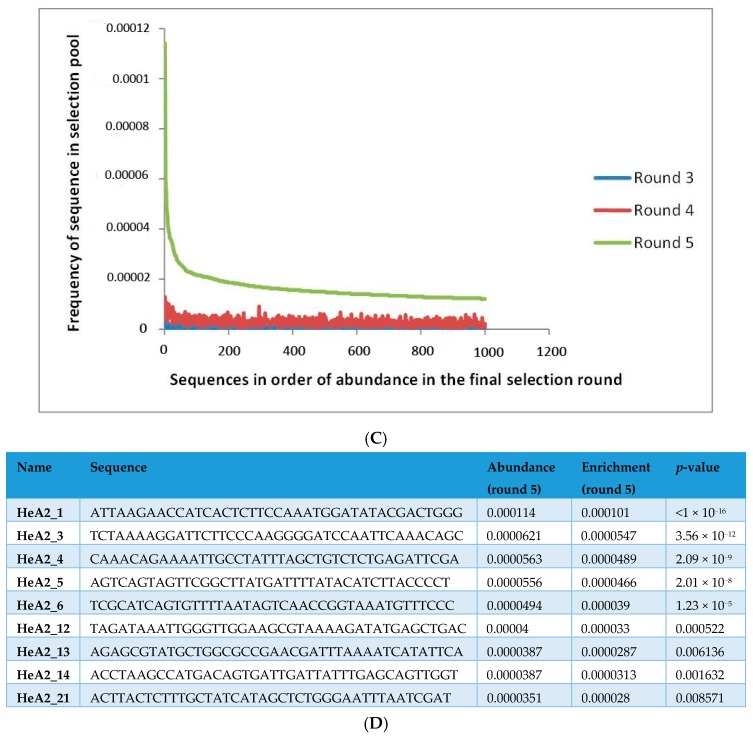
Aptamer selection. (**A**) Schematic overview of the adherent whole-cell SELEX approach; Five selection rounds were performed to enrich DNA aptamer sequences that bind to adherent SKBR3 cells on coverslips (**B**) Progress of the selection analysed by high throughput NGS; (**C**) Abundance of the 1000 most abundant sequences in selection round 5 in selection rounds 3, 4 and 5; (**D**) List of most promising DNA aptamer sequences after five rounds of selection in terms abundance (at round 5), enrichment (from round 4 to 5) and *p*-value. The sequences of the 40-mer random region are listed.

To select aptamers against proteins associated with the cell surface in their natural state, we performed whole-cell SELEX using the adherent SKBR3 breast cancer cell line (Figure 1A). The major advantage of using adherent cells is the ability to easily wash the cells, thereby removing all unbound sequences and those sequences that are (non-specifically) bound to dead or floating cells. To isolate the bound sequences, a combination of treatments was performed, including incubation with 5 mM EDTA for 15 min at 37 °C (to dissociate the cells from the coverslip), followed by treatment with 6 M urea (to denature the cells) and heat treatment for 5 min at 95 °C (to dissociate the aptamer-protein complexes).

In total, five rounds of selection were performed. We sequenced the aptamer pools from each selection round and conducted bioinformatics analysis on a total of 50 million sequences. The progress of the selection was evaluated and the selection was found successful since the heterogeneity of the aptamer pools decreased with the progress of the selection (Figure 1B).

In order to identify candidate aptamers, all individual sequences were analysed based on their frequency within the final aptamer pool (abundance) and the rate at which these frequencies were increasing over multiple selection rounds (enrichment). We believe that the abundance and enrichment of individual sequences are a better predictor of valuable aptamers than conventional motif frequency, as it would appear that selection occurs at the full sequence level rather than at the motif level [42]. Figure 1C shows that the distribution of sequences abundance is not linearly distributed. The top few sequences are present at much higher abundance than the rest of the sequences. Figure 1C also demonstrates that the enrichment rate of the sequences in the final three selection rounds is higher for the very few top sequences. We imposed a statistical filter whereby the sequences must be within the top 1000 sequences in terms of abundance in selection round 5, and the sequences must exhibit *p*-values that are less than 0.01 for enrichment based on comparison of enrichment rates within the top 1000 sequences. This resulted in nine sequences (Figure 1D). The combined total frequency of these nine selected top sequences in the final selection round was only 0.00049. If only 100 sequences would have been analysed (by traditional cloning), the probability of capturing at least one of these sequences would have been 4.9%. This means that there is a 95% probability that, if we had only analysed 100 sequences, we would not have identified any of the sequences that exhibited the strongest response to selection. These results illustrate the significance of NGS and bioinformatics analysis during and after selection.

Since the aptamer structure is of great importance for its functionality, we evaluated the secondary structures of the nine selected aptamers. One single structure was predicted for aptamers HeA2_1, HeA2_3, HeA2_5 and HeA2_6. In contrast, five different structures could be predicted for aptamers HeA2_14 and HeA2_21. The highest structure stability (expressed as Gibbs free energy) was predicted for aptamer HeA2_3 (−9.27 kcal/mol). Amount of structures and structure stability are important aspects for the formulation of therapeutics for reasons of stability and homogeneity.

Another important structure often observed within single-stranded DNA aptamers is the G-quadruplex structure, formed by guanine-rich nucleic acid sequences. It has been demonstrated that G-quadruplexes enhance structure stability, offer increased resistance against nuclease-mediated degradation [43,44] and can be involved in the aptamers functionality [45,46]. We investigated the nine selected aptamers for the presence of G-quadruplexes using QGRS Mapper [47]. However, no G-quadruplexes could be identified.

In whole-cell SELEX, each of the cell surface molecules are a potential target. In our study, we chose HER2 as target of interest. SKBR3 cells are known to overexpress the HER2 protein. The selected aptamers were therefore tested for binding to the HER2 protein and other HER2 overexpressing cell lines.

### 2.2. Aptamer Protein Binding and Binding Affinity 

**Figure 2:**
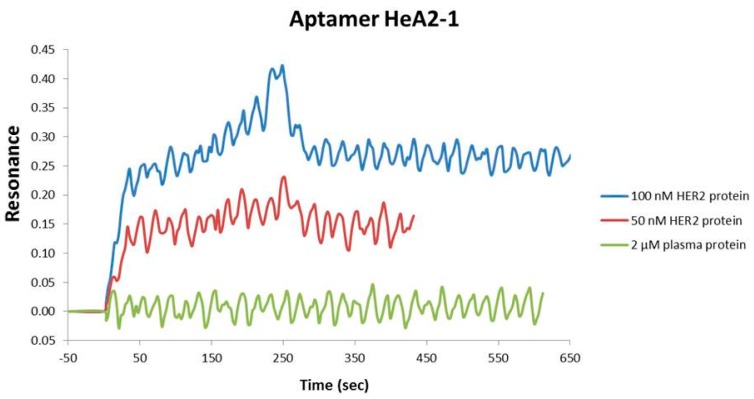

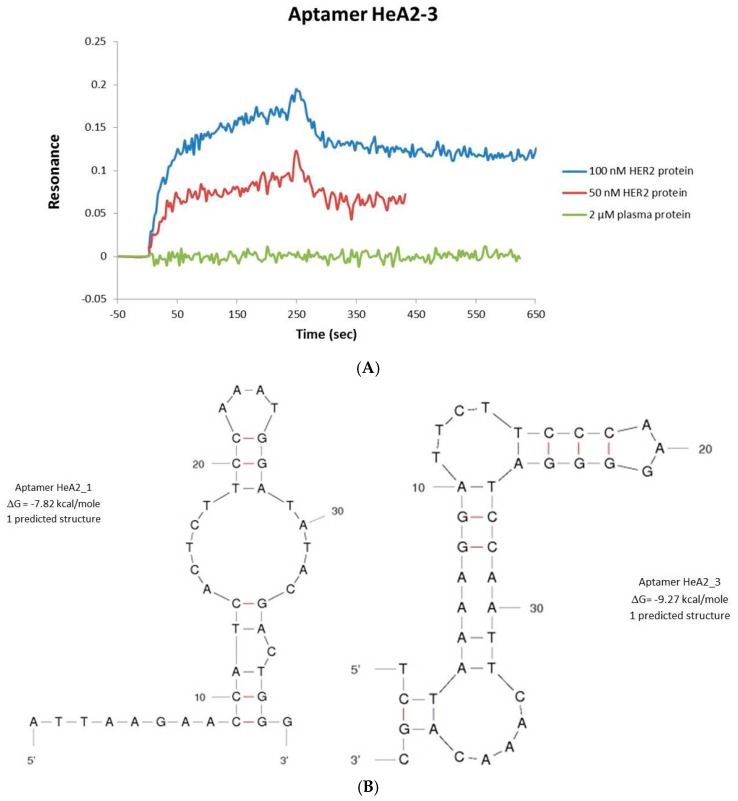
(**A**) Protein binding assays by SPRi. Sensorgrams for aptamer HeA2_1 (**top**) and HeA2_3 (**bottom**). Injection of the protein started at 0 s and ended at 210 s. Data (mean reflexivity units) are representative for 3 biological replicates. The dissociation (Koff) coefficient was calculated as described in the experimental section; (**B**) Predicted aptamer secondary structures. The secondary structure was investigated at conditions on selections (37 °C, 120 mM Na, 5 mM Mg). The secondary structure and Gibbs free energy (ΔG, kcal/mole) were predicted using the Mfold software [49,50].

In order to determine aptamer protein binding and binding affinity, we evaluated the kinetic parameters for aptamer-HER2 complex formation by surface-plasmon resonance imaging (SPRi). The selected aptamers (100 µM) were spotted onto a gold chip. Subsequently, the chip was sequential injected with 100 nM HER2 protein, 2 µM plasma protein and 50 nM HER2 protein. Figure 2 shows the response curves of aptamers HeA2_1 and HeA2_3. Both aptamers have a fast association phase, followed by a slow disassociation phase (Figure 2). No binding was observed for plasma protein. Based on these data, the binding affinity coefficient (Kd) was 28.9 nM for aptamer HeA2_1 and 6.2 nM for aptamer HeA2_3. The calculated Kd values, as well as the Kon and Koff values, are in agreement with the values of other aptamer-protein complexes [48].

### 2.3. Aptamer Specificity for Different Cancer Cell Lines

**Figure 3:**
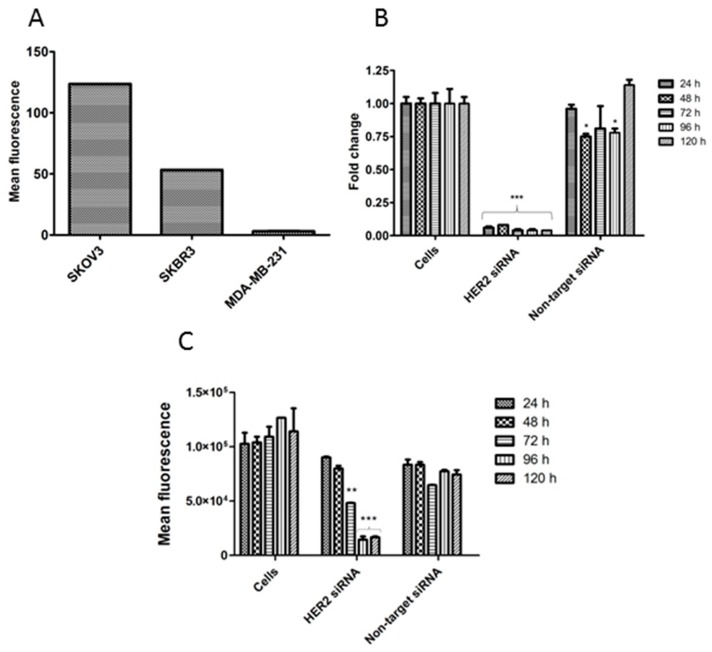
(**A**) HER2 expression level of different cell lines analysed by flow cytometry. Data are expressed as mean ± SD, error bars are smaller than symbols (**B**) HER2 mRNA levels after silencing of SKOV3 cells with HER2-specific siRNA or non-target siRNA. Data are expressed as fold change ± SEM using the Pfaffl method, *n* = 3 biological replicates; (**C**) HER2 protein levels analysed by flow cytometry using anti-HER2 antibody. Results are expressed as mean fluorescence ± SEM, *n* = 2 biological replicates. * *p* < 0.05; ** *p* < 0.01 and *** *p* < 0.001 *vs.* untreated cells, 2-way ANOVA with Bonferroni post-test.

To demonstrate the aptamer specificity, we used cell lines with different levels of HER2 receptors per cell. Since the literature is not consistent in the level of HER2 of these cell lines [51,52,53,54,55,56,57,58,59,60], we investigated the HER2 expression level by flow cytometry using an anti-HER2 antibody (Figure 3A). A 2.3-fold higher HER2 expression could be observed for the SKOV3 cells *vs.* SKBR3 cells and a 40.1-fold difference for SKOV3 *vs.* MDA-MB-231. Moreover, to verify whether the observed aptamer binding to HER2 overexpressing cells was related to HER2, we created SKOV3 cells with low HER2 expression (SKOV3_T) via lipid-based transfection of HER2-specific siRNAs. To confirm silencing, HER2 mRNA and protein levels were determined using Q-PCR and flow cytometry, respectively (Figure 3B,C). As shown in Figure 3B, the HER2 mRNA level was rapidly and significantly decreased after treatment with HER2-specific siRNA and remained constant for the tested period. In contrast, treatment with non-target siRNA showed a variable, but non-specific effect, similar to what we observed when treating cells with the transfecting reagents without siRNA (data not shown). The HER2 protein level was significantly decreased after treatment with HER2-specific siRNA in a gradual way, due to remaining residual HER2 protein (Figure 3C). We observed that the introduction of a second transfection, 72 h after the first transfection, was needed to further reduce the protein level. A maximal reduction of 88.5% could be observed after 96 h. Remarkably, treatment with non-target siRNA also led to a reduction in HER2 protein level with a maximal reduction of 59.1% after 72 h. Together, these data show that the level of HER2 is maximally decreased at 96 h after transfection and that SKOV3_T cells can be used for further HER2-specific assays.

**Figure 4:**
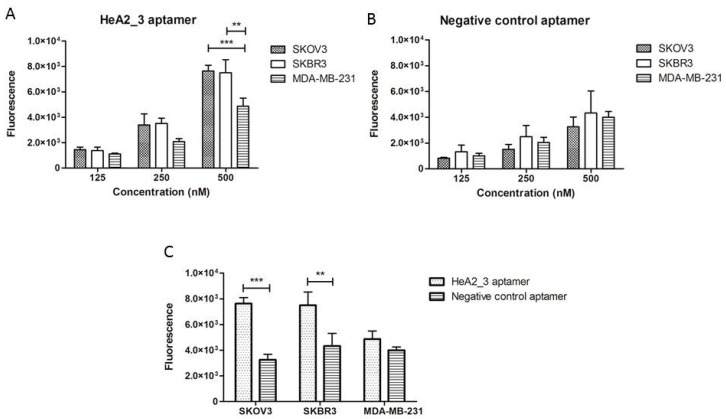
Flow cytometry analysis of aptamer specificity on cells with different HER2 expression levels. Bar graphs represent mean fluorescence of the cells incubated with three different concentrations (125, 250 and 500 nM) aptamer HeA2_3 (**A**) or negative control aptamer (**B**); (**C**) Bar graphs represent mean fluorescence of the cells incubated with 500 nM aptamer HeA2_3 or negative control aptamer. *n* ≥ 3 biological replicates, error bars represent SD, significance of differences was tested using the 2-way ANOVA test with ** *p* < 0.01 and *** *p* < 0.005.

The specificity of aptamer HeA2_3 was evaluated by flow cytometry (Figure 4). Aptamer HeA2_3 revealed a highly significant increase in binding to HER2-overexpressing cells (SKOV3 and SKBR3) compared to cells with low HER2 expression level (MDA-MB-231) at 500 nM concentration (Figure 4A). In contrast, incubation of these cells with a negative control aptamer did not result in a difference in fluorescence between the three cell lines at all tested concentrations (Figure 4B). Moreover, binding of aptamer HeA2_3 was significantly higher on SKOV3 and SKBR3 cells compared to the negative control aptamer (Figure 4C). These results suggest that binding of aptamer HeA2_3 was specific to HER2.

**Figure 5:**
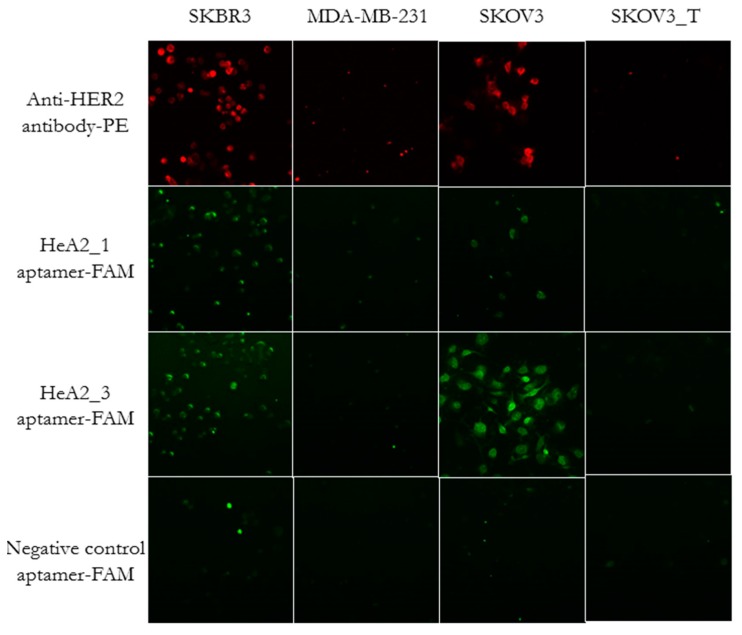
Fluorescence microscopy analysis of the aptamer binding specificity and epitope competition. Cells were stained with both Hoechst (blue, left panels) and anti-HER2 antibody (red, right panels) or aptamers HeA2_1 or HeA2_3 (green, right panels). Images were taken at a magnification of 200×.

We further evaluated the specificity of aptamers HeA2_1 and HeA2_3 by fluorescence microscopy (Figure 5). To avoid possible artefacts from fixation and permeabilization [61], aptamers were incubated on live cells and cells were only fixed after the complete staining procedure (to prevent dehydration during microscopic analysis). Antibody staining confirmed high HER2 expression of SKBR3 and SKOV3 cells and low HER2 expression of MDA-MB-231 cells and transfected SKOV3 cells (SKOV3_T, 96 h). Increased fluorescence intensity of aptamers HeA2_1 and HeA2_3 could be observed for SKBR3 and SKOV3 cells. In contrast, the negative control aptamer did not show staining on the tested cell lines. These data confirm that the selected aptamers bind specifically to HER2 overexpressing cells.

To note, the following experiments were performed with SKOV3 cells instead of SKBR3 cells, because of the higher HER2 level (Figure 3A) and because SKBR3 cells were problematic to grow tumors in mice (data not shown).

### 2.4. Competition for HER2 Binding 

**Figure 6:**
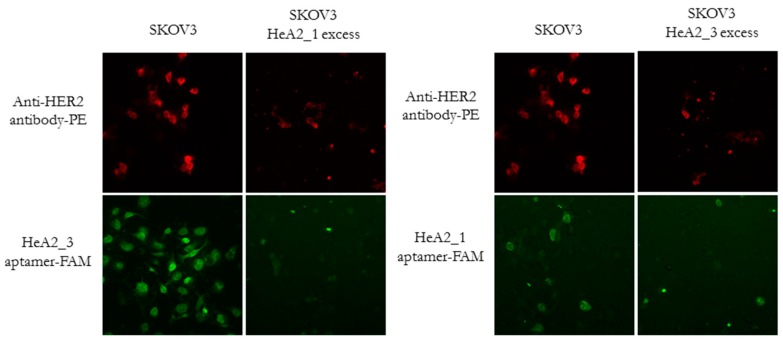
Fluorescence microscopy analysis of the aptamer binding specificity and epitope competition. Cells were stained with the anti-HER2 antibody (red) or aptamers HeA2_1 or HeA2_3 (green) in the absence ((**left**) panels) and presence ((**right**) panels) of an excess aptamer HeA2_1 or HeA2_3. Images were taken at a magnification of 200×.

To evaluate aptamers HeA2_1 and HeA2_3 for competition for HER2 binding and to confirm that binding of the aptamers was HER2-specific, SKOV3 cells were incubated with a 100-fold excess of non-fluorescent aptamer HeA2_1 or HeA2_3 and subsequently stained with the fluorescent anti-HER2 antibody, aptamer HeA2_1 or HeA2_3. As shown in Figure 6, both aptamers were found to specifically bind HER2, since addition of an excess aptamer was able to block the binding of the anti-HER2 antibody.

To evaluate whether the aptamers HeA2_1 and HeA2_3 compete with each other for HER2 binding, SKOV3 cells were incubated with a 100-fold excess of non-fluorescent aptamer HeA2_1 or HeA2_3 and subsequently stained with the fluorescently labeled aptamers HeA2_1 or HeA2_3. We observed less binding after addition of an excess aptamer, suggesting that both aptamers bind HER2 in a competitive manner. We therefore hypothesize that binding should involve the same epitope on HER2 or that HER2 undergoes an allosteric shift as a result of binding, thus changing epitopes. On the other side, it may be possible that the HER2 receptor was unavailable because of internalization after binding.

### 2.5. Internalization of Aptamer HeA2_3 into HER2 Overexpressing Cells

Aptamer-mediated transport of payloads such as toxins, drugs, siRNA or radionuclides, may be more effective when delivered intracellularly. HER2 has no natural ligand but is known to internalize after binding to specific molecules, such as antibodies [62,63,64], nanobodies [65] and affibodies [66,67]. 

**Figure 7:**
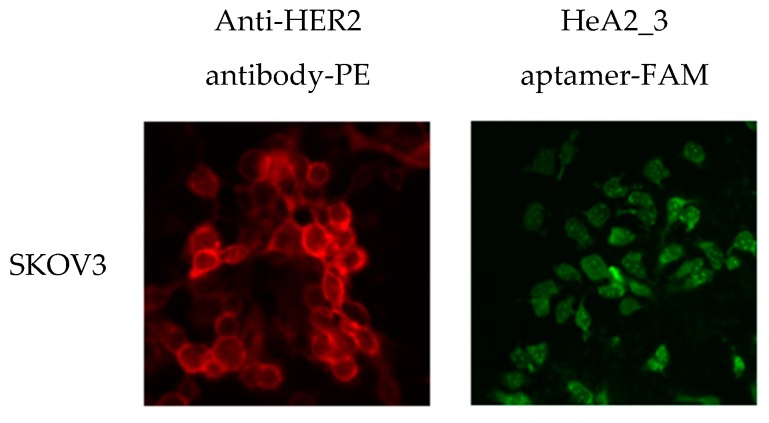
Fluorescence microscopy analysis of internalization of aptamer HeA2_3 into SKOV3 cells. Cultured SKOV3 cells were stained with anti-HER2 antibody (**red**) or HeA2_3 aptamer (**green**) and internalization was evaluated by fluorescence microscopy. Images were taken at a magnification of 400×. All experiments were performed at 37 °C as lower temperatures may inhibit internalization.

To assess whether aptamer HeA2_3 was internalized after HER2 binding, we compared the cellular localization of aptamer HeA2_3 and the anti-HER2 antibody in SKOV3 cells. The antibody displayed the characteristic localization of HER2 at the cell surface, whereas the fluorescent intensity of aptamer HeA2_3 was mostly localized in the cytoplasm (Figure 7). Since the anionic character of oligonucleotides does not favour spontaneous entry into live cells, the intracellular localization of the aptamer suggests that entry into the cells occurred by receptor-mediated endocytosis. In addition, a punctate pattern could be observed, which has also been observed with other internalizing aptamers [40,68,69,70,71,72]. This pattern may indicate aptamer accumulation in the endosomes, which support the hypothesis of receptor-mediated endocytosis as route of internalization [73].

### 2.6. Ex Vivo Tumor Tissue Staining

**Figure 8:**
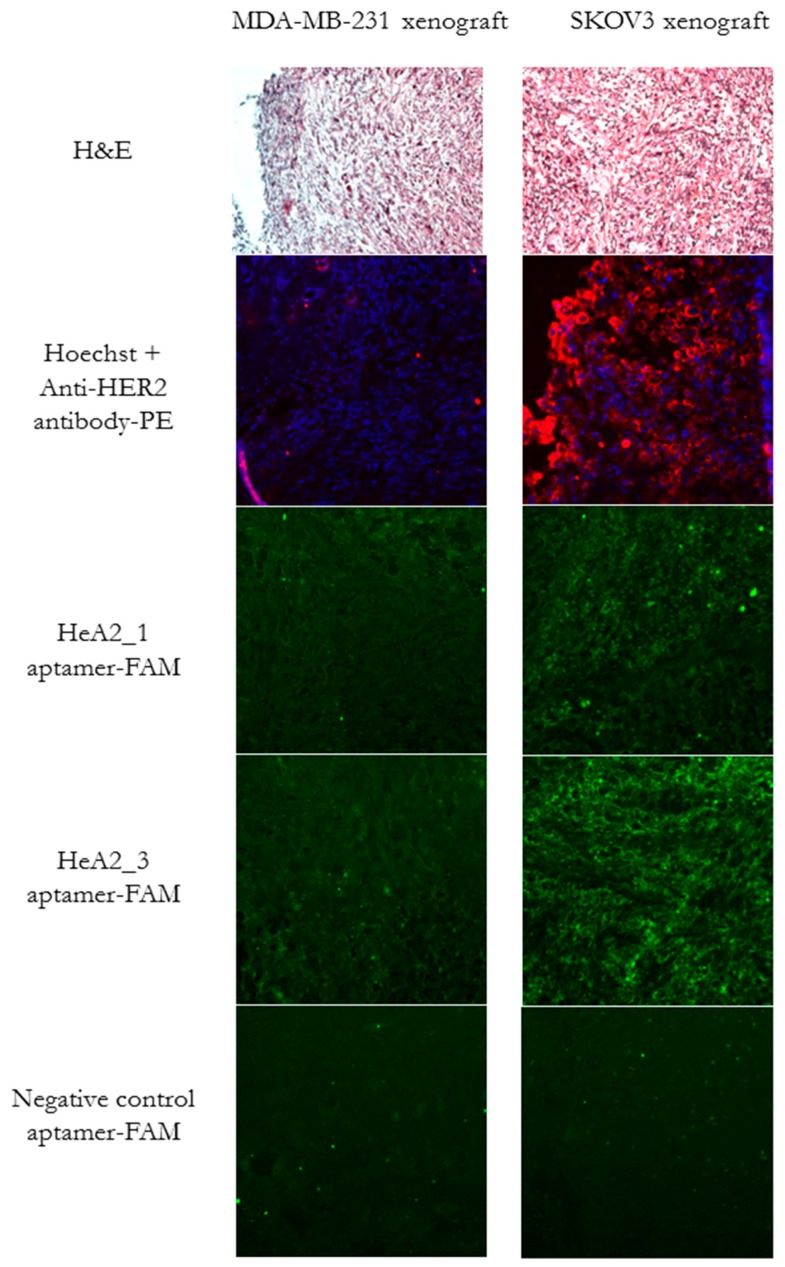
Fluorescence microscopy analysis of aptamer binding to tumor xenografts. Sections of tumor xenografts were stained with hematoxylin and eosin (top), Hoechst (blue) and the anti-HER2 antibody (red) or aptamer HeA2_1, HeA2_3 or the negative control aptamer (green). Images were taken at a magnification of 200×.

Aptamer binding was further evaluated on tumor tissue sections by fluorescence microscopy. For this, immunodeficient mice were inoculated with SKOV3 or MDA-MB-231 cells and tumors were dissected 9 weeks after inoculation. Tumor tissue sections were stained with hematoxylin and eosin for morphology confirmation. A highly dense, poorly differentiated cell mass could be observed (Figure 8). Next, antibody staining confirmed that the HER2 expression of the cell lines was maintained in the xenografts. A bright fluorescent staining could be observed for aptamers HeA2_3 and Hea2_1, although less pronounced for aptamer HeA2_1. No staining was observed for all aptamers on the MDA-MB-231 tumor tissue sections and for the negative control aptamer on both tumor tissue sections. These results confirm specific binding of the aptamers to HER2. In addition, it demonstrates the potential use of aptamers to replace antibodies in immunostaining protocols.

### 2.7. Targeted Inhibition of Cell Growth

The HER2 receptor plays an important role in cell growth and proliferation. Several molecules targeting the HER2 receptor, such as the antibody trastuzumab, showed that binding to HER2 leads to a decrease in cell growth of HER2 overexpressing cells [74,75]. Aptamers have also shown to be able to sterically hinder the active site or interaction surfaces of other protein targets, thereby affecting the function of the targeted protein (reviewed in [76,77]). 

**Figure 9:**
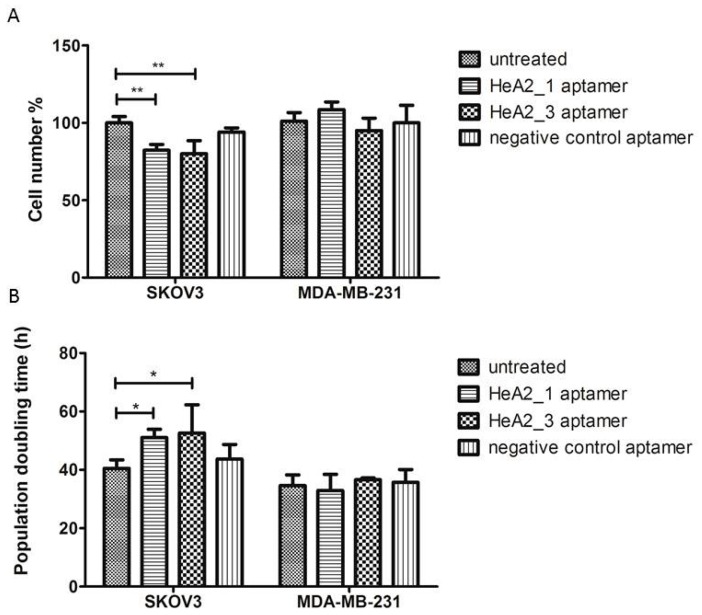
Targeted inhibition of cell growth by selected aptamers. (**A**) Bar graphs represent % cell number after 5 days of daily treatment with or without 0.1 µM aptamer. *n* ≥ 3 biological replicates, error bars represent SD, significance of differences between treated *vs.* untreated cells was tested using the 2-way ANOVA test with ** *p* < 0.01 (**B**) Bar graphs represent overall population doubling time (h) after daily treatment with or without 0.1 µM aptamer. *n* ≥ 3 biological replicates, error bars represent SD, significance of differences between treated *vs.* untreated cells was tested using the 2-way ANOVA test with * *p* < 0.05.

We investigated the potential inhibitory effect of aptamers HeA2_1 and HeA2_3 on cell proliferation of SKOV3 and MDA-MB-231 cells. Cells were treated daily with 0.1 µM aptamer and counted daily up till 5 days after initiation. All cells were analysed in their exponential growth phase. Both HER2 aptamers significantly inhibited the proliferation of SKOV3 cells. As expected, the inhibitory effect was time- and dose-dependent as the largest effect in cell number could be observed at the end of the experiment, which is 5 days after daily treatment, after having received 5 doses of 0.1 µM aptamer. The mean SKOV3 cell number after 5 days of treatment was reduced to 82.2% ± 3.8% and 80.0% ± 8.5%, for aptamers HeA2_1 and HeA2_3 respectively compared to untreated cells (*p* < 0.01) (Figure 9A). In addition, the mean SKOV3 cell number was significantly reduced for aptamer HeA2_3 compared to the negative control aptamer (*p* < 0.05), suggesting that inhibitory effect was related to the aptamer’s specificity for HER2.

The overall population doubling time of SKOV3 cells was significantly increased with 1.26-fold for aptamer HeA2_1 and 1.30-fold for aptamer HeA2_3 compared to untreated cells (*p* < 0.05) (Figure 9B). In case of the MDA-MB-231 cells, treatment with the HER2 aptamers showed no significant effects on cell number, suggesting that the inhibitory effect may be related to the action of HER2. Moreover, no effect on cell proliferation was observed in the presence of the negative control aptamer for both cell lines, suggesting that the tested dose (0.1 µM) was not toxic in a non-specific manner. 

## 3. Materials and Methods

### 3.1. Cell Lines and Cell Culture

Human adherent cell lines SKBR3 (breast adenocarcinoma), MDA-MB-231 (breast adenocarcinoma), SKOV3 (ovarian adenocarcinoma) were purchased from American Type Cell Culture (ATCC, Manassas, VA, USA). SKBR3 and SKOV3 cells were maintained in McCoy’s 5A culture medium (ATCC) supplemented with 20% (*v*/*v*) fetal bovin serum (FBS, Gibco, ThermoFisher Scientific, Gent, Belgium). Both cell lines were cultured in a humidified incubator at 37 °C in the presence of 5% CO_2_ in air. MDA-MB-231 cells were maintained in Leibovitz’s L-15 culture medium (ATCC) supplemented with 10% (*v*/*v*) FBS (Gibco) and 100 Units/mL penicillin and 100 µg/mL streptomycin (Gibco). MDA-MB-231 cells were cultured in a humidified incubator at 37 °C in 100% air. All cell lines were dissociated using 5 mM ethylenediaminetetraacetic acid (EDTA, Sigma-Aldrich, St. Louis, MO, USA).

### 3.2. Transfection

Ten thousand SKOV3 cells were plated in a 24-well plate in antibiotic free medium one day prior transfection. Cells were transfected with 50 or 100 nM ON-TARGETplus SMARTpool Human ERBB2 siRNA (Dharmacon, Lafayette, CO, USA) with 1.5 µL Lipofectamine RNAiMAX reagent (Invitrogen, Carlsbad, CA, USA) in Opti-MEM I Reduced Serum Medium, GlutaMAX (Invitrogen). Complete growth medium was added 4 hours after incubation with the transfection mixture. Cells treated with the transfection reagent alone were used as a reference. In addition, a negative control using 50 or 100 nM ON-TARGET plus non-targeting pool (Dharmacon) was included. Cells were analysed by Q-PCR and flow cytometry for HER2 expression.

### 3.3. HER2 Expression Analysis by Q-PCR (mRNA Level)

RNA isolation (AllPrep DNA/RNA/Protein Mini, Qiagen, Hilden, Germany) and reverse transcription (Taqman Reverse transcription reagens, Applied Biosystems, Foster City, CA, USA) were performed according to manufacturer’s recommendations. Expression levels were evaluated using the TaqMan Gene Expression Assay (Applied Biosystems) following manufacturer’s instructions. Ct values were normalised using GAPDH as endogenous control. Fold changes were calculated using the Pfaffl method. Significance was tested with the two-way ANOVA test.

### 3.4. HER2 Expression Analysis by Flow Cytometry (Protein Level)

Cells were harvested by 5 mM EDTA and stained with the fluorescent anti-human ErbB2-Phycoerythin (PE) monoclonal antibody (R & D Systems, Minneapolis, MN, USA) according to manufacturer’s protocol. As a negative control, cells were stained with a PE-conjugated isotype control antibody (R & D Systems). Cells were analysed using the C6 flow cytometer (Accuri, Ann Arbor, MI, USA). It should be noted that only extracellular HER2 is stained using the PE-conjugated antibody.

### 3.5. Oligonucleotides

The DNA library for selection was composed of a 40-mer random region flanked by two constant regions for primer hybridisation: 5′-TAG GGA AGA GAA GGA CAT ATG AT-(N40)-TTG ACT AGT ACA TGA CCA CTT GA-3′ (Trilink Biotechnologies, San Diego, CA, USA). 

During selection, the eluted sequences were PCR amplified using 1X PCR amplification buffer (New England Biolabs, Ipswich, MA, USA), 2 µM primers and 5 Units/µL Taq polymerase (50 µL total volume). Amplifications were carried out in a MJ Mini Thermal Cycler (BioRad, Hercules, CA, USA) at 94 °C for 10 s, 55 °C for 15 s and 72 °C for 30 s. The appropriate number of PCR cycles was visually determined by 10% acrylamide gel electrophoresis.

After selection, aptamer sequences without primers (40-mer) were chemically synthesized by Sigma Genosys (Oakville, ON, Canada) or Integrated DNA Technologies (IDT, Leuven, Belgium). For fluorescent-based assays, the aptamer sequences were extended with a 25-mer region (5′-TTT TTC CAC AAC GAT GCG TAG TTC CG-3′) complementary to a 20-mer fluorescent probe (5′-FAM-CGG AAC TAC GCA TCG TGT GG-3′). Prior to use, both sequences were combined at an equimolar concentration, heated for 10 min at 95 °C and cooled down to room temperature.

### 3.6. Aptamer Selection

The selection was performed using 20,000 adherent SKBR3 cells which were plated on glass coverslips in a 24-well plate. Once a good lawn of adherent cells was established, the growth medium was removed and replaced by 1 mL of pre-warmed selection buffer (10 mM HEPES, 120 mM NaCl, 5 mM MgCl_2_, 5 mM KCl) containing the random DNA library (10^15^ sequences, corresponding to 1.66 µM). Prior to incubation, the library was heated to 95 °C for 10 min and “snap-cooled” on ice for 15 min. Following an incubation of 30 min at 37 °C, the selection buffer, along with all unbound aptamers and dead cells, was removed and the cells were washed with 1 mL of fresh selection buffer. Next, 500 µL of 5 mM EDTA was applied to the cells and incubated for 15 min at 37 °C to lift the cells from the coverslip. The supernatant containing the cells and the bound aptamers was then added to a urea solution (6 M final concentration) to lyse the cells. In addition, heat treatment was performed (95 °C for 5 min) to dissociate aptamer-protein complexes. The cellular debris was removed by centrifugation at 13,300 rpm for 1 min. The bound aptamers were recovered from the cell lysate using a MinElute PCR Purification Kit (Qiagen). PCR amplification (forward primer 5′-TAG GGA AGA GAA GGA CAT ATG AT-3′, reverse primer 5′-TCA AGT GGT CAT GTA CTA GTC AA-3′) was used to amplify a fraction of the recovered aptamers (10 µL out of 400 µL) for the next round of selection. The PCR products were purified away from the primers with the use of a MinElute PCR Purification Kit (Qiagen). The purified PCR products (400 µL) were diluted with 600 µL selection buffer, heated at 95 °C for 10 min and “snap-cooled” for 15 min, and added to the cells for the next round of selection. In total, 5 rounds of positive selection were performed. A fraction of the eluted aptamer pools from each selection round was sequenced by NGS (Illumina HiSeq 2500, Hospital for Sick Children, Toronto, ON, Canada). For each sequence, a copy number was observed in the NGS data. This copy number was normalized to a frequency (or abundance) by dividing it by the total number of sequences observed for that selection round. Enrichment refers to the increase in frequency from one selection round to another. We determined *Z*-values (observed value—mean value)/standard deviation. Next, we determined *p*-values for these *Z*-values using the Excel function 1-NORMSDIST (*Z*-value). The secondary structures of the selected sequences were predicted using mfold software at the conditions of selection (37 °C, 120 mM Na^+^ and 5 mM Mg^2+^) [49,50]. In addition, the selected sequences were analysed for potential quadruplex forming G-rich sequences by QGRS Mapper [47,78].

### 3.7. Protein Binding by Surface Plasmon Resonance Imaging (SPRi)

Two aptamers were chosen for further investigation: HeA2_3: 5′-TCT AAA AGG ATT CTT CCC AAG GGG ATC CAA TTC AAA CAG C-3′ and HeA2_1: 5′-ATT AAG AAC CAT CAC TCT TCC AAA TGG ATA TAC GAC TGG G-3′. A negative control aptamer of the same sequence length was also used (5′-CCC TTT TAC ACA ACC ATC GAC ATA ACT AAA ACC ACC ACT G-3′). 

The aptamers were synthesized with hexyl-disulfide group on the 5′ end and spotted (10 nL, 100 µM) in triplicate on a gold sensor chip. The chip was blocked with a coating of bovine serum albumin (Fischer, Ried im Innkreis, Austria). The chip was loaded on top of a prism in an OpenPlex SPRi instrument (Horiba Scientific, Edison, NJ, USA). HER2 protein (50 or 100 nM final concentration) (monomer, extracellular domain, Met1-Thr652, Sino Biological Inc., North Wales, PA, USA) and plasma protein (2 µM final concentration) were diluted in selection buffer and injected at a volume of 200 µL and a flow rate of 50 µL/min into the instrument. Bound protein was removed with 1 M NaCl (4 min). The chip was calibrated using 3 mg/mL sucrose (Sigma Aldrich). 

Resonance (R) was measured using the optical sensor of the instrument. An increase in resonance observed when the protein flows over the chip, is a function of the protein binding to the aptamers. Data was analysed by subtracting the resonance values observed as the protein flows over the negative aptamer spots from the resonance values observed as the protein passes over the HeA2_1 and HeA2_3 aptamer spots. 

Data were analysed in R using the ‘Neo-Bind’ program (NeoVentures Biotechnology Inc.). The coefficient of disassociation (Koff) was determined by the following formula: *dR*/*dt* = – *Koff* × *Rt* (dR/dt = the first derivative of the resonance values, and *Rt* = the resonance value for each time point evaluated). The following formula was used to determine the coefficient of association (Kon): *dR*/*dt* = – *Kon* × *c* × *Rmax* – (*Konn × c* + *Koff*) × *Rt* (*c* = protein concentration in the flow, *Rmax* = the maximal observed resonance). The binding affinity coefficient (*Kd*) was calculated by dividing *Koff* by *Kon*.

### 3.8. Flow Cytometry

Cells were detached from the culture flask using non-enzymatic dissociation buffer (5 mM EDTA in PBS) at 37 °C for 10 min. Dissociated cells (100,000) were washed in selection buffer and incubated with varying concentrations (125, 250 and 500 nM) of aptamer HeA2_3 or negative control aptamer in selection buffer at 37 °C for 60 min. After incubation, unbound aptamers were removed by centrifugation and the cells were washed with PBS. Cells were kept on ice prior to flow cytometry analysis on an Accuri C6 flow cytometer (BD Bioscience, San Jose, CA, USA). The resulting mean fluorescence was subtracted with the mean (auto)fluorescence of untreated cells.

### 3.9. Fluorescent Microscopy—Cellular Staining

Cells (20,000 cells in 500 µL culture medium) were seeded in LabTek II 4-well chamber slides (Nunc, Sigma Aldrich, St. Louis, MO, USA) and cultured for 4 to 5 days. The cells were carefully washed with PBS and then incubated with the aptamers at a final concentration of 250 nM in selection buffer. After incubation (30 min at 37 °C), cells were washed with PBS to remove unbound aptamers. For antibody staining, cells were blocked with human IgG (R & D Systems) for 15 min at room temperature. Afterwards, 10 µL of monoclonal anti-HER2 antibody (R & D Systems) was added and incubated for 30 min at 37 °C. Hoechst staining was performed by incubating the cells with 0.1 mg/mL Hoechst final concentration (Sigma Aldrich) for 10 min at room temperature. Finally, cells were fixed using 4% sucrose in 4% paraformaldehyde (PFA) for 10 min at room temperature. Images were acquired with a 40× objective on a Eclipse Ti automated inverted fluorescence microscope (Nikon Instruments Inc., Melville, NY, USA) equipped with bandpass filters for Hoechst (excitation 387/11, emission 452/45), FAM (excitation 485/20, emission 536/40) and PE (excitation 556/20, emission 593/40). Differential interference contrast (DIC) images are bright field. All images were taken with the same exposure time. For competition studies, a 100-fold of non-fluorescent aptamer HeA2_1 or HeA2_3 was added to SKOV3 cells for 30 min at 37 °C. Next, unbound excess aptamer was removed and fluorescent antibody or aptamer was added to the cells and incubated for 30 min at 37 °C. Cells were counterstained with Hoechst as mentioned above. Fluorescent images were not always merged with DIC or Hoechst for reasons of clarity.

### 3.10. Fluorescent Microscopy—Tumor Tissue Staining

#### 3.10.1. Animals

Hairless non-obese diabetic severe combined immunodeficient (NOD.Cg-Prkdc^scid^Hr^hr^/NCrHsd) mice were purchased from Harlan Laboratories/Envigo (Indianapolis, IN, USA). Mice were housed together (3–4 mice per cage) in individual ventilated cages and were kept under controlled conditions of 12:12 h light:dark cycle, 22 ± 2 °C and 50% ± 5% relative humidity. The mice were fed ad libitum with irradiated rodent food (Teklad, Harlan Laboratories) and autoclaved water. The animals were housed at the animal facility of SCK•CEN in accordance with the Ethical Committee Animal Studies of Medanex Clinic (EC_MxCl_2014_034). All animal experiments were done in compliance with the NIH Guides for the Care and Use of Laboratory Animals and were approved by the Ethical Committee of the University of Liège. Mice were inoculated subcutaneously in the hind leg with a 100 µL cell suspension composed of 50 µL cells (3 × 10^6^ MDA-MB-231 or 1.5 × 10^6^ SKOV3) and 50 µL Matrigel basement membrane matrix high concentration (Corning, Corning, NY, USA).

#### 3.10.2. *Ex Vivo* Tumor Tissue Staining

Xenografted tumors were harvested, washed with PBS and incubated overnight in 4% PFA at 4 °C. The tumors were subsequently washed three times with PBS (5 min) and incubated in 10% sucrose (2 h), 20% sucrose (2 h) and 30% sucrose (overnight) at 4 °C. Next, the tumors were placed in liquid optimal-cutting-temperature medium Tissuetek (Sakura, AJ Alphen aan den Rijn, the Netherlands) and flash-frozen for storage at −20 °C. Frozen tumor tissue sections were cut (10 µm thick) with a CryoStar™ NX50 Cryostat (Thermo Scientific, Waltham, MA, USA). Hematoxylin and eosin staining was performed for morphological confirmation following routine laboratory protocol. For antibody staining, the tumor tissue sections were blocked with human IgG (R & D Systems) for 15 min at room temperature. Afterwards, 10 µL of monoclonal anti-HER2 antibody (R & D Systems) was added and incubated for 30 min at 37 °C. Hoechst staining was performed by incubating the tumor tissue sections with 0.1 mg/mL Hoechst final concentration (Sigma Aldrich) for 10 min at room temperature. For aptamer staining, the tumor tissue sections were washed with PBS and incubated with the aptamer at a final concentration of 250 nM in selection buffer. After incubation (30 min at 37 °C), the tumor tissue sections were washed with PBS to remove unbound aptamers. Images were acquired as described above.

### 3.11. Cell Proliferation Assay

Cells were seeded in a T25 culture flask (100,000 cells in 3 mL culture medium) and cultured. After 24 h, cells were treated daily with 0.1 µM aptamer (HeA2_1, HeA2_3 or negative control). Fresh culture medium was added on day 2, 4 and 6. At various time points, cells were dissociated with 5 mM EDTA and counted using the MOXI Z mini automated cell counter (curve fit count mode, VWR). Population doubling time (h) was extrapolated from modelling the data with the exponential growth equation y = y_0_^(k*x)^ with y = cell number at time x, y_0_ = initial cell number, k = rate constant) (GraphPad). All samples were determined in triplicate.

## 4. Conclusions

In this study, we selected novel DNA aptamers targeting the HER2 receptor, an important protein in cancer development and progression and thus an attractive target for cancer therapy. In contrast to the previously selected HER2 aptamers, the aptamers selected in this study are relatively short (40-mer). This short length avoids the need for laborious truncation experiments and facilitates a more efficient synthesis, as it is known that the synthesis efficiency decreases by 3% per nucleotide. Moreover, the synthesis can be more inexpensive, which is important when translating aptamers to diagnostic and therapeutic applications. 

These aptamers were selected using an adherent whole-cell SELEX approach using the cells in their most natural state. Over the last decade, several aptamers have been developed using the whole-cell SELEX approach on living cells, such as bacteria, immune cells and cancer cells (reviewed in [79,80,81,82]). However, most of these approaches are complex, laborious and time-consuming. We designed a protocol including high throughput NGS and bioinformatics analysis instead of traditional cloning and subsequent sequencing. This approach allowed us to reduce the number of iterative selection rounds, to only select high affinity sequences and to minimize the introduction of related sequences from PCR artefacts [25,32,33,83]. This idea was supported by the report of Schütze *et al.* who demonstrated that most clones that occur after round 5 are derivatives of strongly enriched clones, which can be attributed to either mutation or sequencing artefacts [31,83]. 

Affinity and specificity of binding are two of the most important characteristics for aptamers in oncology. High affinity is crucial for aptamers for efficient targeting and long tumor residence time. The aptamers selected in this study have affinities in the low nanomolar range. However, high affinity (<10 nM) is not always needed as it was demonstrated that a too high affinity restricts efficient tumor penetration (so-called binding side barrier hypothesis) [63,84,85,86,87]. It should also be mentioned that the affinity was estimated based on the kinetics of the aptamer-protein complex formation by SPRi, which may not reflect the *in vivo* (cellular) situation. In particular, because we showed that aptamer HeA2_3 was internalized (and probably subsequently degraded in the endosomes), the dissociation constant (*Koff*) becomes pointless. Moreover, *Kd* values highly depend on the experimental method used and must therefore be interpreted with caution [48].

Specificity may be of more value than affinity, as the specificity will drive both the potency of the aptamer and its side effect profile. Therefore, we focused on demonstrating the specificity of the aptamers selected in this study. To accomplish this, we set up binding assays using a variety of cell lines, a transfected cell line and tumor tissue sections with different expression levels of HER2. One of the best ways to actually visualize aptamer binding is by fluorescent microscopy. Our major findings showed that the selected aptamers bind HER2-overexpressing cells in a highly specific and competitive manner. These results show that both aptamers possess excellent targeting properties and are therefore good candidates as tools for *in vitro* diagnostics. In addition, they can also be used for *in vivo* diagnostic applications, after coupling to a detectable moiety, such as diagnostic radionuclides or contrast agents.

Interestingly, aptamer HeA2_3 was able to be internalized into HER2-overexpressing cancer cells, which may be useful for the aptamer-mediated delivery of toxic payloads, such as drugs, siRNA and radionuclides. Moreover, we showed that aptamers HeA2_1 and HeA2_3 possessed an extra desirable feature for therapeutic applications because it retarded cell growth. We hypothesize that the internalization of HER2 after aptamer binding may be involved in this growth retardation, because internalization of receptors results in both short and long term loss of receptor activity and because HER2-overexpressing cancer cells are highly dependent on HER2 signaling for survival and proliferation [88,89,90,91].

In conclusion, our findings open up new possibilities for the development of a novel promising therapeutic aptamer. In a next step, the *in vivo* therapeutic potential of this aptamer should be investigated. Finally, its ability to replace (or complement) the existing HER2-targeting therapeutics, by overcoming their drawbacks such as treatment resistance and cardiotoxicity, will need to be demonstrated. In the end, this will provide new opportunities in personalized medicine, in particular in the area of targeted cancer therapy.

## References

[1] Slamon D.J., Clark G.M., Wong S.G., Levin W.J., Ullrich A., McGuire W.L. (1987). Human breast cancer: Correlation of relapse and survival with amplification of the HER-2/neu oncogene. Science.

[2] Slamon D.J., Godolphin W., Jones L.A., Holt J.A., Wong S.G., Keith D.E., Levin W.J., Stuart S.G., Udove J., Ullrich A. (1989). Studies of the HER-2/neu proto-oncogene in human breast and ovarian cancer. Science.

[3] Zhang J., Liu Y. (2008). HER2 over-expression and response to different chemotherapy regimens in breast cancer. J. Zhejiang Univ. Sci. B.

[4] Musolino A., Ciccolallo L., Panebianco M., Fontana E., Zanoni D., Bozzetti C., Michiara M., Silini E.M., Ardizzoni A. (2011). Multifactorial central nervous system recurrence susceptibility in patients with HER2-positive breast cancer: Epidemiological and clinical data from a population-based cancer registry study. Cancer.

[5] Rouanet P., Roger P., Rousseau E., Thibault S., Romieu G., Mathieu A., Cretin J., Barneon G., Granier M., Maran-Gonzalez A. (2014). HER2 overexpression a major risk factor for recurrence in pT1a-bN0M0 breast cancer: Results from a French regional cohort. Cancer Med..

[6] Fight HER2+ Breast Cancer with Herceptin. http://www.herceptin.com.

[7] Bartsch R., Wenzel C., Steger G.G. (2007). Trastuzumab in the management of early and advanced stage breast cancer. Biologics.

[8] Claret F.X., Vu T.T. (2012). Trastuzumab: Updated mechanisms of action and resistance in breast cancer. Front. Oncol..

[9] Sengupta P.P., Northfelt D.W., Gentile F., Zamorano J.L., Khandheria B.K. (2008). Trastuzumab-induced cardiotoxicity: Heart failure at the crossroads. Mayo Clin. Proc..

[10] Ulrich H., Erdmann V., Barciszewski J., Brosius J. (2006). RNA aptamers: From basic science towards therapy. RNA towards Medicine.

[11] Bouchard P.R., Hutabarat R.M., Thompson K.M. (2010). Discovery and development of therapeutic aptamers. Annu. Rev. Pharmacol. Toxicol..

[12] Hu M., Zhang K. (2013). The application of aptamers in cancer research: An up-to-date review. Future Oncol..

[13] Barbas A.S., Mi J., Clary B.M., White R.R. (2010). Aptamer applications for targeted cancer therapy. Future Oncol..

[14] Bruno J.G. (2013). A review of therapeutic aptamer conjugates with emphasis on new approaches. Pharmaceuticals.

[15] Meyer C., Hahn U., Rentmeister A. (2011). Cell-specific aptamers as emerging therapeutics. J. Nucleic Acids.

[16] Thiel K.W., Giangrande P.H. (2009). Therapeutic applications of DNA and RNA aptamers. Oligonucleotides.

[17] Keefe A.D., Pai S., Ellington A. (2010). Aptamers as therapeutics. Nat. Rev. Drug Discov..

[18] Weinberg M.S. (2014). Therapeutic aptamers march on. Mol. Ther. Nucleic Acids.

[19] Sundaram P., Kurniawan H., Byrne M.E., Wower J. (2013). Therapeutic RNA aptamers in clinical trials. Eur. J. Pharm. Sci..

[20] Tuerk C., Gold L. (1990). Systematic evolution of ligands by exponential enrichment: RNA ligands to bacteriophage T4 DNA polymerase. Science.

[21] Ellington A.D., Szostak J.W. (1990). *In vitro* selection of RNA molecules that bind specific ligands. Nature.

[22] Robertson D.L., Joyce G.F. (1990). Selection *in vitro* of an RNA enzyme that specifically cleaves single-stranded DNA. Nature.

[23] Graham J.C., Zarbl H. (2012). Use of cell-selex to generate DNA aptamers as molecular probes of HPV-associated cervical cancer cells. PLoS ONE.

[24] Penner G. Dubbles, an Alternative to Selex. https://www.youtube.com/watch?v=rmpvqCX1WqA.

[25] Blind M., Blank M. (2015). Aptamer selection technology and recent advances. Mol. Ther. Nucleic Acids.

[26] Pan W., Xin P., Patrick S., Dean S., Keating C., Clawson G. (2010). Primer-free aptamer selection using a random DNA library. J. Vis. Exp..

[27] Pan W., Clawson G.A. (2009). The shorter the better: Reducing fixed primer regions of oligonucleotide libraries for aptamer selection. Molecules.

[28] Marimuthu C., Tang T.H., Tominaga J., Tan S.C., Gopinath S.C. (2012). Single-stranded DNA (ssDNA) production in DNA aptamer generation. Analyst.

[29] Le L.C., Cruz-Aguado J.A., Penner G.A. (2011). DNA Ligands for Aflatoxin and Zearalenone. U.S. Patent.

[30] Penner G. Detection Systems. http://neoventures.ca/products/mycotoxin-testing/.

[31] Schütze T., Wilhelm B., Greiner N., Braun H., Peter F., Mörl M., Erdmann V.A., Lehrach H., Konthur Z., Menger M. (2011). Probing the SELEX process with next-generation sequencing. PLoS ONE.

[32] Cho M., Xiao Y., Nie J., Stewart R., Csordas A.T., Oh S.S., Thomson J.A., Soh H.T. (2010). Quantitative selection of DNA aptamers through microfluidic selection and high-throughput sequencing. Proc. Natl. Acad. Sci. USA.

[33] Dausse E., Taouji S., Evade L., di Primo C., Chevet E., Toulme J.J. (2011). HAPIscreen, a method for high-throughput aptamer identification. J. Nanobiotechnol..

[34] Thiel W.H., Bair T., Thiel K.W., Dassie J.P., Rockey W.M., Howell C.A., Liu X.Y., Dupuy A.J., Huang L., Owczarzy R. (2011). Nucleotide bias observed with a short SELEX RNA aptamer library. Nucleic Acid Ther..

[35] Kang H.-S., Huh Y.-M., Kim S., Lee D.K. (2009). Isolation of RNA aptamers targeting HER2-overexpressing breast cancer cells using cell-SELEX. Bull. Korean Chem. Soc..

[36] Kim M.Y., Jeong S. (2011). *In vitro* selection of RNA aptamer and specific targeting of ErbB2 in breast cancer cells. Nucleic Acid Ther..

[37] Thiel K.W., Hernandez L.I., Dassie J.P., Thiel W.H., Liu X., Stockdale K.R., Rothman A.M., Hernandez F.J., McNamara J.O., Giangrande P.H. (2012). Delivery of chemo-sensitizing siRNAs to HER2+-breast cancer cells using RNA aptamers. Nucleic Acids Res..

[38] Gupta S., Thirstrup D., Jarvis T.C., Schneider D.J., Wilcox S.K., Carter J., Zhang C., Gelinas A., Weiss A., Janjic N. (2011). Rapid histochemistry using slow off-rate modified aptamers with anionic competition. Appl. Immunohistochem. Mol. Morphol..

[39] Liu Z., Duan J.-H., Song Y.-M., Ma J., Wang F.-D., Lu X., Yang X.-D. (2012). Novel HER2 aptamer selectively delivers cytotoxic drug to HER2-positive breast cancer cells *in vitro*. J. Transl. Med..

[40] Mahlknecht G., Maron R., Mancini M., Schechter B., Sela M., Yarden Y. (2013). Aptamer to ErbB-2/HER2 enhances degradation of the target and inhibits tumorigenic growth. Proc. Natl. Acad. Sci. USA.

[41] Hu Y., Duan J., Cao B., Zhang L., Lu X., Wang F., Yao F., Zhu Z., Yuan W., Wang C. (2015). Selection of a novel DNA thioaptamer against HER2 structure. Clin. Transl. Oncol..

[42] Ozer A., Pagano J.M., Lis J.T. (2014). New technologies provide quantum changes in the scale, speed, and success of SELEX methods and aptamer characterization. Mol. Ther. Nucleic Acids.

[43] Bishop J.S., Guy-Caffey J.K., Ojwang J.O., Smith S.R., Hogan M.E., Cossum P.A., Rando R.F., Chaudhary N. (1996). Intramolecular G-quartet motifs confer nuclease resistance to a potent anti-HIV oligonucleotide. J. Biol. Chem..

[44] Casals J., Viladoms J., Pedroso E., Gonzalez C. (2010). Structure and stability of a dimeric G-quadruplex formed by cyclic oligonucleotides. J. Nucleic Acids.

[45] Breaker R.R. (1997). DNA aptamers and DNA enzymes. Curr. Opin. Chem. Biol..

[46] Tucker W.O., Shum K.T., Tanner J.A. (2012). G-quadruplex DNA aptamers and their ligands: Structure, function and application. Curr. Pharm. Des..

[47] Kikin O., D’Antonio L., Bagga P.S. (2006). QGRS mapper: A web-based server for predicting G-quadruplexes in nucleotide sequences. Nucleic Acids Res..

[48] Cload S.T., McCauley T.G., Keefe A.D., Healy J.M., Wilson C. (2006). Properties of therapeutic aptamers. The Aptamer Handbook.

[49] Zuker M. (2003). Mfold web server for nucleic acid folding and hybridization prediction. Nucleic Acids Res..

[50] The Unafold Web Server. http://mfold.rna.albany.edu/.

[51] DeFazio-Eli L., Strommen K., Dao-Pick T., Parry G., Goodman L., Winslow J. (2011). Quantitative assays for the measurement of HER1-HER2 heterodimerization and phosphorylation in cell lines and breast tumors: Applications for diagnostics and targeted drug mechanism of action. Breast Cancer Res..

[52] Tolmachev V. (2008). Imaging of HER2 overexpression in tumors for guiding therapy. Curr. Pharm. Des..

[53] Subik K., Lee J.F., Baxter L., Strzepek T., Costello D., Crowley P., Xing L., Hung M.C., Bonfiglio T., Hicks D.G. (2010). The expression patterns of ER, PR, HER2, CK5/6, EGFR, Ki-67 and AR by immunohistochemical analysis in breast cancer cell lines. Breast Cancer.

[54] Savinainen K.J., Saramaki O.R., Linja M.J., Bratt O., Tammela T.L., Isola J.J., Visakorpi T. (2002). Expression and gene copy number analysis of ErbB2 oncogene in prostate cancer. Am. J. Pathol..

[55] Hermanto U., Zong C.S., Wang L.H. (2001). ErbB2-overexpressing human mammary carcinoma cells display an increased requirement for the phosphatidylinositol 3-kinase signaling pathway in anchorage-independent growth. Oncogene.

[56] Ginestier C., Adelaide J., Goncalves A., Repellini L., Sircoulomb F., Letessier A., Finetti P., Geneix J., Charafe-Jauffret E., Bertucci F. (2007). ErbB2 phosphorylation and trastuzumab sensitivity of breast cancer cell lines. Oncogene.

[57] Magnifico A., Albano L., Campaner S., Delia D., Castiglioni F., Gasparini P., Sozzi G., Fontanella E., Menard S., Tagliabue E. (2009). Tumor-initiating cells of HER2-positive carcinoma cell lines express the highest oncoprotein levels and are sensitive to trastuzumab. Clin. Cancer Res..

[58] Cuello M., Ettenberg S.A., Clark A.S., Keane M.M., Posner R.H., Nau M.M., Dennis P.A., Lipkowitz S. (2001). Down-regulation of the ErbB-2 receptor by trastuzumab (herceptin) enhances tumor necrosis factor-related apoptosis-inducing ligand-mediated apoptosis in breast and ovarian cancer cell lines that overexpress ErbB-2. Cancer Res..

[59] Govindarajan S., Sivakumar J., Garimidi P., Rangaraj N., Kumar J., Rao N., Gopal V. (2011). Targeting human epidermal growth factor receptor 2 by a cell-penetrating peptide-affibody bioconjugate. Biomaterials.

[60] Lewis G., Figari I., Fendly B., Wong W., Carter P., Gorman C., Shepard H. (1993). Differential responses of human tumor cell lines to anti-p185HER2 monoclonal antibodies. Cancer Immunol. Immunother..

[61] Schnell U., Dijk F., Sjollema K.A., Giepmans B.N. (2012). Immunolabeling artifacts and the need for live-cell imaging. Nat. Methods.

[62] Austin C.D., de Maziere A.M., Pisacane P.I., van Dijk S.M., Eigenbrot C., Sliwkowski M.X., Klumperman J., Scheller R.H. (2004). Endocytosis and sorting of ErbB2 and the site of action of cancer therapeutics trastuzumab and geldanamycin. Mol. Biol. Cell.

[63] Rudnick S.I., Lou J., Shaller C.C., Tang Y., Klein-Szanto A.J., Weiner L.M., Marks J.D., Adams G.P. (2011). Influence of affinity and antigen internalization on the uptake and penetration of anti-HER2 antibodies in solid tumors. Cancer Res..

[64] De Goeij B.E., Peipp M., de Haij S., van den Brink E.N., Kellner C., Riedl T., de Jong R., Vink T., Strumane K., Bleeker W.K. (2014). HER2 monoclonal antibodies that do not interfere with receptor heterodimerization-mediated signaling induce effective internalization and represent valuable components for rational antibody-drug conjugate design. MAbs.

[65] Pruszynski M., Koumarianou E., Vaidyanathan G., Revets H., Devoogdt N., Lahoutte T., Lyerly H.K., Zalutsky M.R. (2014). Improved tumor targeting of anti-HER2 nanobody through *N*-succinimidyl 4-guanidinomethyl-3-iodobenzoate radiolabeling. J. Nucl. Med..

[66] Wallberg H., Orlova A. (2008). Slow internalization of anti-HER2 synthetic affibody monomer 111In-DOTA-ZHER2:342-pep2: Implications for development of labeled tracers. Cancer Biother. Radiopharm..

[67] Ahlgren S., Orlova A., Wallberg H., Hansson M., Sandstrom M., Lewsley R., Wennborg A., Abrahmsen L., Tolmachev V., Feldwisch J. (2010). Targeting of HER2-expressing tumors using 111In-ABY-025, a second-generation affibody molecule with a fundamentally reengineered scaffold. J. Nucl. Med..

[68] Xiao Z., Levy-Nissenbaum E., Alexis F., Lupták A., Teply B.A., Chan J.M., Shi J., Digga E., Cheng J., Langer R. (2012). Engineering of targeted nanoparticles for cancer therapy using internalizing aptamers isolated by cell-uptake selection. ACS Nano.

[69] Chen C.H., Dellamaggiore K.R., Ouellette C.P., Sedano C.D., Lizadjohry M., Chernis G.A., Gonzales M., Baltasar F.E., Fan A.L., Myerowitz R. (2008). Aptamer-based endocytosis of a lysosomal enzyme. Proc. Natl. Acad. Sci. USA.

[70] Porciani D., Signore G., Marchetti L., Mereghetti P., Nifosi R., Beltram F. (2014). Two interconvertible folds modulate the activity of a DNA aptamer against transferrin receptor. Mol. Ther. Nucleic Acids.

[71] Veldhoen S., Laufer S.D., Restle T. (2008). Recent developments in peptide-based nucleic acid delivery. Int. J. Mol. Sci..

[72] Gourronc F.A., Rockey W.M., Thiel W.H., Giangrande P.H., Klingelhutz A.J. (2013). Identification of RNA aptamers that internalize into HPV-16 E6/E7 transformed tonsillar epithelial cells. Virology.

[73] Lodish H., Berk A., Kaiser C.A., Krieger M., Scott M.P., Bretscher A., Ploegh H., Matsudaira P. (2000). Molecular Cell Biology.

[74] Brockhoff G., Heckel B., Schmidt-Bruecken E., Plander M., Hofstaedter F., Vollmann A., Diermeier S. (2007). Differential impact of Cetuximab, Pertuzumab and Trastuzumab on BT474 and SK-BR-3 breast cancer cell proliferation. Cell Prolif..

[75] Kim S.Y., Kim H.P., Kim Y.J., Oh do Y., Im S.A., Lee D., Jong H.S., Kim T.Y., Bang Y.J. (2008). Trastuzumab inhibits the growth of human gastric cancer cell lines with HER2 amplification synergistically with cisplatin. Int. J. Oncol..

[76] Famulok M., Hartig J., Mayer G. (2007). Functional aptamers and aptazymes in biotechnology, diagnostics, and therapy. Chem. Rev..

[77] Kaur G., Roy I. (2008). Therapeutic applications of aptamers. Expert Opin. Investig. Drugs.

[78] QGRS Mapper. http://bioinformatics.ramapo.edu/QGRS/index.php.

[79] Ohuchi S. (2012). Cell-SELEX technology. BioRes. Open Access.

[80] Zhang Y., Chen Y., Han D., Ocsoy I., Tan W. (2010). Aptamers selected by cell-SELEX for application in cancer studies. Bioanalysis.

[81] Sefah K., Shangguan D., Xiong X., O’Donoghue M.B., Tan W. (2010). Development of DNA aptamers using cell-SELEX. Nat. Protoc..

[82] Ye M., Hu J., Peng M., Liu J., Liu J., Liu H., Zhao X., Tan W. (2012). Generating aptamers by cell-SELEX for applications in molecular medicine. Int. J. Mol. Sci..

[83] Thiel W.H., Bair T., Peek A.S., Liu X., Dassie J., Stockdale K.R., Behlke M.A., Miller F.J., Giangrande P.H. (2012). Rapid identification of cell-specific, internalizing rna aptamers with bioinformatics analyses of a cell-based aptamer selection. PLoS ONE.

[84] Juweid M., Neumann R., Paik C., Perez-Bacete M.J., Sato J., van Osdol W., Weinstein J.N. (1992). Micropharmacology of monoclonal antibodies in solid tumors: Direct experimental evidence for a binding site barrier. Cancer Res..

[85] Fujimori K., Covell D.G., Fletcher J.E., Weinstein J.N. (1990). A modeling analysis of monoclonal antibody percolation through tumors: A binding-site barrier. J. Nucl. Med..

[86] Van Osdol W., Fujimori K., Weinstein J.N. (1991). An analysis of monoclonal antibody distribution in microscopic tumor nodules: Consequences of a “binding site barrier”. Cancer Res..

[87] Adams G.P., Schier R., McCall A.M., Simmons H.H., Horak E.M., Alpaugh R.K., Marks J.D., Weiner L.M. (2001). High affinity restricts the localization and tumor penetration of single-chain fv antibody molecules. Cancer Res..

[88] Witsch E., Sela M., Yarden Y. (2010). Roles for growth factors in cancer progression. Physiology.

[89] Fink M.Y., Chipuk J.E. (2013). Survival of HER2-positive breast cancer cells: Receptor signaling to apoptotic control centers. Genes Cancer.

[90] Iqbal N., Iqbal N. (2014). Human epidermal growth factor receptor 2 (HER2) in cancers: Overexpression and therapeutic implications. Mol. Biol. Int..

[91] Neve R.M., Lane H.A., Hynes N.E. (2001). The role of overexpressed HER2 in transformation. Ann. Oncol..

